# Potential Factors Associated With Commercial-to-Medicare Relative Prices at the Substate Level

**DOI:** 10.1001/jamahealthforum.2025.1640

**Published:** 2025-07-03

**Authors:** Fredric Blavin, John Holahan

**Affiliations:** 1 Health Policy Division, Urban Institute, Washington, DC

## Abstract

**Importance:**

There is a growing consensus that commercial prices vary in ways that do not reflect quality of care and are a key factor in high health care spending in the US.

**Objective:**

To assess the geographic variation in commercial prices relative to Medicare rates for both hospital and professional services at the state and substate levels, estimate the change in these prices and determine which characteristics are associated with higher hospital prices.

**Design, Setting, and Participants:**

This cross-sectional study analyzed deidentified aggregated health care claims data for 2 time frames of service, from January 1, 2020, through December 31, 2020, and from June 1, 2022, through May 31, 2023, to construct commercial-to-Medicare price ratios for hospital and professional services at the state and geozip levels (491 geozips correspond to combinations of zip codes in 50 states and the District of Columbia). Multivariable regression models were estimated to assess the association between commercial-to-Medicare relative hospital prices and various market characteristics at the geozip level. Data analysis was conducted from July through November 2024.

**Exposures:**

Exposures defined at the geozip level included hospital and insurer market concentrations, the share of hospitals beds associated with nonprofit hospitals, the share of beds associated with health systems, the presence of a major teaching hospital, mean household income, the share of the population who had public health insurance, and the share who were uninsured.

**Main Outcomes and Measures:**

Commercial prices relative to Medicare rates for inpatient, outpatient, combined hospital, and professional services.

**Results:**

This cross-sectional study of 1.2 billion claim lines in 2020 and 1.5 billion claim lines from June 2022 through May 2023 found that private insurers’ in-network allowed amounts were 246% (ratio [SD], 2.46 [0.6]) of the Medicare rates for hospital services and 124% (ratio [SD], 1.24 [0.3]) of the Medicare rates for professional services. The mean commercial-to-Medicare price ratio for professional services slightly declined from 2020 to 2022-2023, while the mean (SD) price ratio for hospital services increased by 5.5%, from 2.34 (0.5) in 2020 to 2.46 (0.6) in 2022-2023. There was substantial variation in the commercial-to-Medicare price ratios across states and geozips. Geozips with very high hospital market concentration levels (Herfindahl-Hirschman Index [HHI]>3500) were associated with a commercial-to-Medicare price ratio higher by 0.21 (95% CI, 0.02-0.39; *P* = .03) relative to geozips with HHI levels lower than 1500, which represents an 8.4% increase above the 2022-2023 mean. High insurer concentration was negatively associated with the commercial-to-Medicare hospital price ratios (−0.13; 95% CI, −0.26 to 0.01; *P* = .04), whereas having a major teaching hospital in the geozip (0.20; 95% CI, 0.06-0.34; *P* = .01), being in the highest household income quartile (0.35; 95% CI, 0.13-0.57; *P* = .002), and the share of the population who were uninsured (0.03; 95% CI, 0.01-0.05; *P* < .001) were positively associated with price ratios.

**Conclusions and Relevance:**

Examination of a major claims database revealed substantial geographic variation in commercial-to-Medicare price ratios and increases in the price ratio for hospital services over time. Substate market and hospital characteristics were also associated with higher commercial-to-Medicare relative prices. These factors, including high hospital market concentration, could be used to identify and target specific areas more amenable to policies aimed at curbing hospital price growth.

## Key Points

**Question:**

How do commercial prices relative to Medicare rates vary across markets; how have these prices changed from 2020 to June 2022 through May 2023; and which characteristics are associated with higher hospital prices?

**Findings:**

This cross-sectional study of 1.2 billion claim lines in 2020 and 1.5 billion claim lines from June 2020 through May 2023 found that commercial in-network allowed amounts were 246% of Medicare rates for hospital services and 124% for professional services, with substantial geographic variation. Higher commercial-to-Medicare price ratios were associated with high hospital market concentration, lower insurer concentration levels, presence of a major teaching hospital, and higher share of the population who were uninsured.

**Meaning:**

Results of this study suggest that prices that health systems negotiate with commercial payers vary in ways that can be attributable to market characteristics.

## Teaser

This cross-sectional study examines claims data by geographic areas to identify features associated with higher and lower insurance-allowed costs relative to Medicare rates and potential contributors to rising heath care prices.

## Introduction

Between 2000 and 2020, average annual prices in the US for medical care grew at nearly twice the rate (4.9% per year) as prices for goods and services in the economy (2.5%), with substantially higher growth for hospital services (10.2%).^1^ Although personal health care price growth slowed to 2% to 3% from 2020 to 2023 during the COVID-19 pandemic, the Centers for Medicare & Medicaid Services (CMS) projects that health care price growth will return to rates near longer-term averages and will exceed economy-wide projections through 2032.^2,3^

There is a growing consensus that commercial prices vary in ways that do not reflect quality of care and are a key factor in high health care spending in the US.^4,5,6,7^ While some of this price growth could reflect improvements in health care quality, various studies have found that market concentration and increasing vertical and horizontal consolidation of hospitals and physician offices into ever larger health care systems have exacerbated higher prices.^8,9,10,11,12,13^ Insurer competition and its interplay with hospital concentration also affect hospital prices. For example, if insurers have considerable market power relative to hospitals, they can negotiate lower reimbursement rates and prices, whereas in a competitive insurance market, hospitals can refuse to deal with an aggressive insurer. In contrast, higher concentration can also increase overall health care costs by giving insurers more leverage in charging higher premiums, which in turn could allow them to be less aggressive in using their market power in negotiating rates with hospitals.^14,15^ Empirical evidence also suggests that insurer market concentration is associated with lower hospital prices, with mixed evidence on the extent to which insurers’ pricing power varies across different levels of hospital consolidation.^9,16^

In this study, we analyzed aggregated deidentified data from a large geographically representative private insurance claims database (the FAIR Health National Private Insurance Claims repository; hereafter, the database or repository) to assess (1) the geographic variation in commercial in-network allowed amounts relative to Medicare rates for both hospital and professional services at the state and substate levels; (2) how these rates changed from calendar year 2020 to June 2022 through May 2023; and (3) which market, hospital, and population characteristics are associated with higher hospital rates. We primarily used data drawn from the database, which holds more than 49 billion private health care claim records for medical and dental services in all areas of the US from 2002 to the present.^17^

## Methods

This cross-sectional study was exempted from review by the Urban Institute’s institutional review board because it did not involve human participants. The study design and reporting followed the Strengthening the Reporting of Observational Studies in Epidemiology (STROBE) reporting guideline for cross-sectional studies.

### Insurance Claims Database

This analysis used aggregated deidentified health care claims data compiled and maintained by a neutral source of robust data and data tools (Fair Health). This study focused on 2 time frames defined by dates of services, from January 1, 2020, through December 31, 2020, and from June 1, 2022, through May 31, 2023. We refer to this second time frame as the 2022-2023 period. The database is made up of deidentified data from billions of privately billed claims for medical and dental services submitted by health care professionals to health insurers across all 50 states, the District of Columbia, Puerto Rico, and the US Virgin Islands. The database currently includes more than 49 billion billed procedures, from 2002 to the present; more than 3 billion new claim records are received each year. The data are contributed by more than 75 health plans, insurance carriers, and third-party administrators and include actual negotiated or in-network allowed amounts from both self-insured and fully insured plans.^17^ The repository has been determined by CMS to be statistically reliable in all 50 states and the District of Columbia and has been used by researchers for various purposes.^18,19,20,21,22,23^

The database provided actual in-network negotiated rates for specific *Current Procedural Terminology* (*CPT*) and Medicare Severity Diagnosis Related Group (MS-DRG) grouper codes in 491 geographic areas (known as geozips) in 50 states and the District of Columbia that generally correspond to combinations of 3-digit zip codes. For both hospital outpatient and professional services, we received data on the number of claims, the median commercial in-network allowed amount, the mean commercial in-network allowed amount, and the Medicare rate for the top 45 professional and outpatient *CPT* codes (determined by 2020 frequency and expenditure nationwide in the in the repository) for each geozip, representing approximately half of the professional and outpatient spending in the database. Those procedures were then fixed across the geographic areas and time periods specific to this study. Professional Medicare rates were based on the associated Medicare Physician Fee Schedule value for the year, *CPT* code, and geographic area. Outpatient Medicare rates were calculated based on the associated Outpatient Prospective Payment System rate for the year, *CPT* code, and geographic areas. For inpatient services, we received similar data for MS-DRG codes in 490 geozips in the US (data were missing for 1 geozip in Alaska). Inpatient Medicare rates were calculated based on the associated Inpatient Prospective Payment System rate for the year, MS-DRG, and geographic area, excluding hospital-specific adjustments.

The database relies on actual allowed amounts for all its medical professional, facility, and Healthcare Common Procedure Coding System–allowed benchmark products. The database no longer uses an imputation method for any of these products. The database implemented these changes due to the broader industry shift toward price transparency and reliance on actual allowed amounts in claims adjudication at the state and federal levels.

Within each geozip and state, we then calculated the ratio of the median commercial price to the Medicare price for each *CPT* and MS-DRG code and generated expenditure-weighted means across the professional, outpatient, and inpatient service codes. The expenditure weights were generated by multiplying the mean commercial price in the geozip by the claim frequency for a specific code. We also created a combined hospital service price variable at the state and geozip levels using the expenditure-weighted distribution across inpatient and outpatient services.

### Other Data Sources

We used 2022 data from the American Hospital Association Annual Survey Database to construct the Herfindahl-Hirschman Index (HHI), a measure of market concentration.^24^ We first calculated the HHI at the hospital referral region (HRR) level.^25^ The HHI is calculated by squaring the market share (based on adjusted admissions) of each unique health system and independent hospital competing within each HRR and then summing the resulting numbers. HHI values can range from 0 to 10 000, where values closer to 0 indicate more a competitive market and a value of 10 000 indicates that a market is controlled by a single health system. We restricted the sample to hospitals providing general medical and surgical services and excluded federal hospitals. We then matched each HRR to the zip code and ultimately geozip level using the crosswalk and population weights from a geographic correspondence engine (Geocorr 2022; MCDC Data Applications).^26^ The geozip-level HHI measure was a weighted mean of the HHIs that had any overlap with the geozip.

We also used the 2022 American Hospital Association data to construct the total number of hospitals beds and number of hospital beds associated with major teaching hospitals, nonprofit hospitals, and health systems for each zip code. We considered major teaching hospitals to be all hospitals that had the Council of Teaching Hospitals designation. We crosswalked each zip code to the geozip level using the population weights from the geographic correspondence engine and estimated the share of hospital beds within each geozip that were part of major teaching hospitals, nonprofit hospitals, and health systems.

Finally, we used the 2017-2022 five-year estimates from the American Community Survey to the construct mean household income and the health insurance distribution (share with public insurance, private insurance, and no insurance) at the zip code tabulation area (ie, generalized geographic representation of a zip code used for statistical purposes) level. These measures were also aggregated to the geozip level using the population weights from the geographic correspondence engine.

We used 2023 combined data on total product market HHI (ie, preferred provider organization, health maintenance organization, point of service, and exchange) for 382 metropolitan statistical areas (MSAs) in models that controlled for health insurer concentration. These data were published by the American Medical Association and come from the Decision Resources Group Managed Market Surveyor.^27^ Similar to our approach for the hospital HHI data, we first matched each MSA to the zip code and ultimately geozip level using the crosswalk and population weights from geographic correspondence engine. The geozip-level insurer HHI measure was a weighted mean of the MSAs that had any overlap with the geozip. However, this measure was limited because it was only available for MSAs and not micropolitan or nonmetro areas; for example, 8 geozips (approximately 2% of all geozips) did not include any population living in an MSA, and 63 geozips (13%) had less than half of their population living in MSAs. As such, our models with the health insurer HHI variables were limited to geozips where any of the population lived in an MSA. As a sensitivity test, we also estimated a model where at least 75% of the population lived in an MSA.

### Statistical Analysis

We used linear regressions at the geozip level to estimate the associations between the 2022-2023 commercial-to-Medicare price ratio for hospital services and an HHI hospital categorical variable (HHI<1500, 1500-2500, 2500-3500, or ≥3500), a high insurer concentration indicator (HHI>2000), the share of hospitals beds associated with nonprofit hospitals, the share of beds associated with health systems, an indicator for having a major teaching hospital in the geozip, quartile indicators for mean household income, the share of the population who had public health insurance, and the share who were uninsured. We conducted statistical tests using robust SEs and used 2-tailed hypothesis testing with a significance threshold of *P* < .05, unless otherwise indicated. The statistical analysis was performed from July through November 2024, using Stata, version 17 (StataCorp).

## Results

### Changes in Commercial-to-Medicare Price Ratios, 2020 to 2022-2023

In this study of 1.2 billion claim lines in 2020 and 1.5 billion claim lines from June 2020 through May 2023, the mean commercial in-network allowed amount for professional services declined by 2.2%, from 127% (ratio [SD], 1.27 [0.3]) of the Medicare fee in 2020 to 124% (ratio [SD], 1.24 [0.3]) in 2022-2023, while the commercial price for in-patient and outpatient hospital services increased by 5.5%, from 234% (ratio [SD], 2.34 [0.5]) of the Medicare fee in 2020 to 246% (ratio [SD], 2.46 [0.6]) in 2022-2023 (Table 1). The increase in the commercial-to-Medicare price ratio for hospital services was due to both increases in the ratio for outpatient services and a larger share of hospital expenditures attributable to outpatient services in 2022-2023. The commercial-to-Medicare price ratio for outpatient services increased from 3.77 in 2020 to 3.83 in 2022-2023 (1.5%), whereas the price ratio for inpatient services (ratio [SD], 1.90 [0.3] in 2020 and ratio [SD], 1.89 [0.3] in 2022-2023) did not change. Moreover, the share of hospital expenditures attributable to outpatient services, with a mean commercial-to-Medicare price ratio twice that of inpatient services, increased from 23% in 2020 to 29% in 2022-2023.

**Table 1.** Commercial-to-Medicare Price Ratios, 2020 to 2022-2023^a^

Service type	Price ratio (SD)		Change, %
	2020	2022-2023	
Professional services	1.27 (0.3)	1.24 (0.3)	−2.2
Combined hospital services	2.34 (0.5)	2.46 (0.6)	5.5
Outpatient services	3.77 (0.9)	3.83 (0.9)	1.5
Inpatient services	1.90 (0.3)	1.89 (0.3)	0.0

^a^ Source: FAIR Health National Private Insurance Claims repository.^17^ Time frames defined by dates of services, from January through December 2020 and from June 2022 through May 2023.

The price ratios in Table 1 do not show the variation in price changes at the state level and the mean Medicare reimbursement rate by service type. For example, the mean commercial-to-Medicare price ratio for professional services declined in all but 4 states (California, Massachusetts, District of Columbia, and Delaware), whereas the mean hospital price ratio increased in 33 states (including District of Columbia) and declined in 18 states (eTable in Supplement 1). In addition, the mean Medicare reimbursement rate increased by less than 1% for professional services, increased by 5% for outpatient services, and declined by 6% for inpatient services (data not shown).

### Geographic Variation in Prices

**Figure 1.**
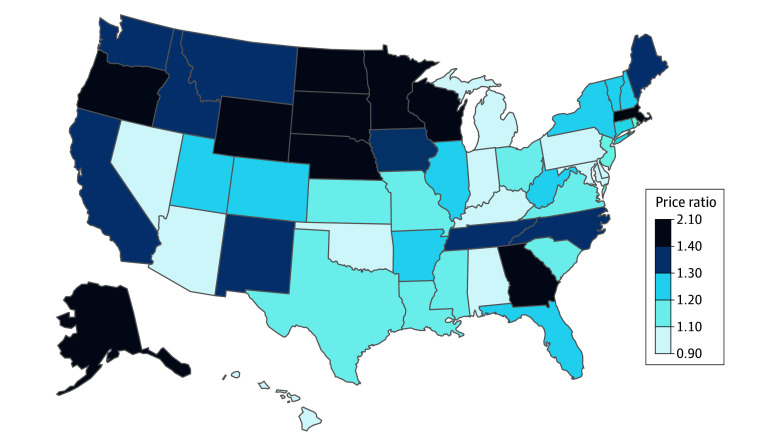
Commercial-to-Medicare Price Ratio for Professional Services by State, 2022-2023 Time frame defined by dates of services from June 2022 through May 2023. Source: FAIR Health National Private Insurance Claims repository.^17^

Figure 1 highlights the differences across states in the commercial-to-Medicare price ratio for professional services in 2022-2023. The national mean for the professional service price ratio was 1.24, ranging from 0.91 in Alabama to 2.09 in Alaska. Rounding out the top 5 highest-priced states were Minnesota (2.06), Wisconsin (1.97), Wyoming (1.80), and North Dakota (1.76). The next 4 lowest-cost states, all of which had mean commercial prices for professional services just below Medicare levels, include Arizona (0.96), Maryland (0.98), Pennsylvania (0.98), and Indiana (0.99).

**Figure 2.**
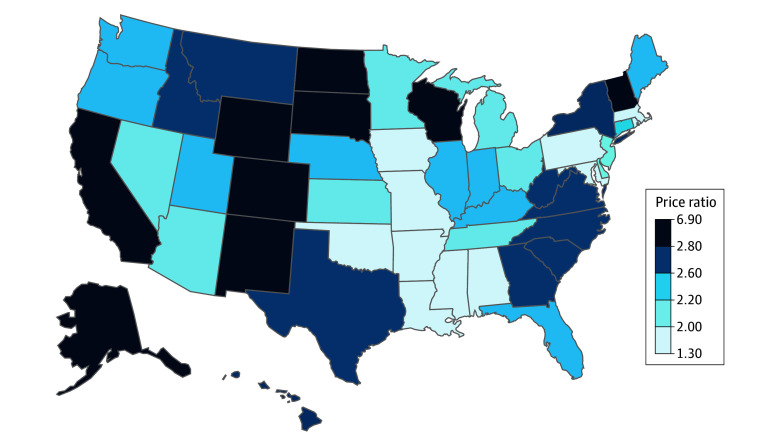
Commercial-to-Medicare Price Ratio for Combined Hospital Services by State, 2022-2023 Time frame defined by dates of services from June 2022 through May 2023. Source: FAIR Health National Private Insurance Claims repository.^17^

Figure 2 shows considerably more variation in the commercial-to-Medicare price ratio for hospital services in 2022-2023. The national mean for the hospital price ratio was 2.46, ranging from 1.28 in Arkansas to 6.90 in Vermont. The next 4 states with the highest commercial-to-Medicare hospital price ratios included Alaska (5.00), Wyoming (3.70), South Dakota (3.60), and Colorado (3.50), while Mississippi (1.45), Massachusetts (1.46), Alabama (1.49), and Maryland (1.69) had the lowest price ratios.

The maps in eFigures 1 and 2 in Supplement 1 highlight within-state variation in price ratios at the geozip level in 2023-2023. For professional services (eFigure 1 in Supplement 1), there was a large concentration of geozips in the highest price quintile in the upper Midwest. For example, all geozips in Minnesota, North Dakota, and Wisconsin were in the top price quintile, with commercial-to-Medicare price ratios ranging from 1.40 to 3.00. In addition, all geozips in Nebraska, South Dakota, Alaska, Wyoming, Iowa, Idaho, Maine, Montana, and Oregon were in the highest or second-highest price quintile for professional services. In contrast, there was substantial price variation for professional services across geozips in the rest of the country. For hospital services (eFigure 2 in Supplement 1), there was a concentration of geozips in the lower price quintiles in Alabama, Mississippi, Louisiana, Arkansas, and eastern Oklahoma, with higher-priced geozips concentrated in the Mountain and West North Central regions.

### Regression Analysis

Table 2 includes the regression results, where the dependent variable was the 2022-2023 commercial-to-Medicare price ratio for hospital services. The hospital price ratio was weighted more toward inpatient services, since 71% of hospital expenditures in 2022-2023 were associated with inpatient services and 29% were associated with outpatient services.

**Table 2.** Association Between Commercial-to-Medicare Price Ratio for Combined Hospital Services and Geozip Characteristics, 2022-2023^a^

Variable	Regression model specification (95% CI)		
	No insurer controls	Insurer HHI	
		>0% Geozip population in an MSA	>75% Geozip population in an MSA
High insurer HHI>2000	NA	−0.13 (−0.26 to 0.01)	−0.20 (−0.36 to 0.04)
Hospital HHI			
1500-2499	0.14 (−0.05 to 0.32)	0.13 (−0.05 to 0.31)	0.12 (−0.09 to 0.32)
2500-3499	0.12 (−0.07 to 0.32)	0.13 (−0.06 to 0.32)	0.17 (−0.05 to 0.38)
≥3500	0.21 (0.02-0.39)	0.22 (0.04-0.40)	0.19 (−0.01 to 0.39)
Beds in geozip, %			
Nonprofit	0.00 (−0.00 to 0.00)	0.00 (−0.00 to 0.00)	−0.00 (−0.00 to 0.00)
System	−0.00 (−0.01 to 0.00)	−0.00 (−0.01 to 0.00)	−0.00 (−0.00 to 0.00)
Any teaching hospital in geozip	0.20 (0.06-0.34)	0.23 (0.09-0.37)	0.27 (0.12-0.42)
Insurance status, % of population			
Uninsured	0.03 (0.01-0.05)	0.03 (0.01-0.05)	0.03 (0.02-0.05)
Public coverage	0.00 (−0.01 to 0.01)	0.00 (−0.01 to 0.01)	−0.01 (−0.02 to 0.01)
Household income quartile			
Second	0.11 (−0.05 to 0.27)	0.13 (−0.03 to 0.29)	0.11 (−0.09 to 0.31)
Third	0.18 (−0.01 to 0.36)	0.21 (0.02-0.39)	0.18 (−0.04 to 0.40)
Fourth (highest)	0.35 (0.13-0.57)	0.39 (0.17-0.61)	0.34 (0.08-0.60)
Constant	1.67 (1.19-2.15)	1.68 (1.20-2.15)	1.87 (1.29-2.45)
Observations, No.	490	483	361
*R* ^2^	0.07	0.08	0.12

Abbreviations: HHI, Herfindahl-Hirschman Index; MSA, metropolitan statistical area; NA, not applicable.

^a^ Source: FAIR Health National Private Insurance Claims repository.^17^ Time frame defined by dates of services from June 2022 through May 2023. All models were estimated using robust SEs.

Factors positively associated with the commercial-to-Medicare price ratio included very high hospital HHI (>3500), having a major teaching hospital in the geozip, mean household income, and the share of the population who were uninsured. In the full sample model without insurer HHI controls, geozips with very high market concentration levels were associated with a commercial-to-Medicare price ratio higher by 0.21 (95% CI, 0.02-0.39; *P* = .03) relative to geozips with HHI levels under 1500, which represented an 8.4% increase above the national mean of 2.46 in 2022-2023. Having a major teaching hospital in the geozip had a similar magnitude (0.20; 95% CI, 0.06-0.34; *P* = .01) to the very HHI indicator, whereas a geozip in the highest mean household income quartile was associated with a commercial-to-Medicare price ratio higher by 0.35 (95% CI, 0.13-0.57; *P* = .002) relative to a geozip in the lowest income quintile (or 14.2% relative to the mean). Finally, a 1 percentage point increase in the uninsured rate was associated with an increase in the relative price ratio of 0.03 (95% CI, 0.01-0.05; *P* < .001), or 1.2% relative to the 2022-2023 mean.

A negative association was found between health insurer concentration and hospital prices in both the samples that include geozips with any population in an MSA and those that include at least 75% of the population in an MSA (Table 2). We found that geozips with health insurer market concentration levels greater than 2000 were associated with commercial-to-Medicare price ratios lower by 0.13 (−0.13; 95% CI, −0.26 to 0.01; *P* = .04) to 0.20 (−0.20; 95% CI, −0.36 to 0.04; *P* = .01) relative to geozips with HHI levels under 2000, which represents a 5% to 8% decrease compared with the national mean in 2022-2023. The coefficients associated with the hospital HHI measures and other controls were similar across all 3 models.

Table 3 presents 3 market characteristics that were associated with higher commercial-to-Medicare hospital price ratios: geozips with very high levels of hospital market concentration, geozips that had a major teaching hospital, and geozips in the highest mean household income quartile. Geozips with very high levels of market concentration were smaller (in terms of population) and more than twice as likely to be classified as rural (26.9% vs 13.1%) and to be in the lowest income quartile (27.7% vs 11.9%) than geozips with low market concentration. In contrast, geozips that had a teaching hospital were more populous, more likely to be urban (91.1% vs 69.6%), and less likely to be in the lowest mean income quartile (17.8% vs 28.1%) than geozips without a major teaching hospital. A similar pattern also held for the highest income vs lowest income geozips.

**Table 3.** Characteristics of Geozips Based on Hospital Market Concentration, Presence of Teaching Hospital, and Mean Household Income Quartile, 2022-2023^a^

Characteristic	HHI		Teaching hospital		Income quartile	
	Very high, >3500	Low, <1500	Yes	No	Highest	Lowest
2020 population	579 300	729 733	857 924	595 122	841 024	500 131
Urban, %	73.1	86.9	91.1	69.6	97.5	49.6
Rural, %	26.9	13.1	8.9	30.4	2.5	50.4
Mean household income quartile, %						
1 (Lowest)	27.7	11.9	17.8	28.1	0.0	100.0
2	29.2	11.9	26.0	24.6	0.0	0.0
3	26.9	33.3	29.5	23.2	0.0	0.0
4 (Highest)	16.2	42.9	26.7	24.1	100.0	0.0
Very high HHI, %	100	0	30.1	24.9	17.2	29.3
With teaching hospital, %	33.8	38.1	100	0	32.0	21.1

Abbreviation: HHI, Herfindahl-Hirschman Index.

^a^ Source: FAIR Health National Private Insurance Claims repository,^17^ American Hospital Association Annual Survey Database, and American Community Survey. Time frame defined by dates of services from June 2022 through May 2023.

## Discussion

In 2022-2023, commercial in-network allowed amounts were 246% of the Medicare fees for hospital inpatient and outpatient services and 124% of the Medicare fees for professional services, with substantial variation both across and within states. In addition, the commercial-to-Medicare price ratio was significantly higher for outpatient services (3.83) than for inpatient services (1.89).

These levels are generally consistent with findings from other health care databases that have different data contributors and sample sizes. For example, RAND estimated a price ratio of 2.54 for combined hospital services in Round 5 (2022) of its Employer-Led Transparency Initiative.^28^ In a review of studies between 2010 and 2020, the Congressional Budget Office (CBO) found that hospital prices paid by commercial insurers were more than twice those paid by the Medicare Fee-for-Service (FFS) program (223%, on average), with the ratio of commercial insurers’ prices to Medicare FFS prices being much higher for outpatient services than for inpatient services. CBO also found that commercial insurers’ prices were on average 129% of Medicare FFS prices for physician/professional services.^29^ The relatively modest changes in the commercial allowed amount data during this period are also consistent with the CMS analysis of national health care spending in 2022, which found low levels of price growth for hospitals (2.8%) and physician and clinical services (0.5%).^30^ The major contributions of our study, however, are that our price data were available at the substate level, and we were able to assess the association between key market characteristics and prices.

Our multivariable findings are also consistent with those of several studies^8,9,10,11,12,13,16^ reporting that health system concentration is associated with higher hospital prices and insurer concentration is associated with lower price ratios. We found that markets with very high hospital concentration levels were more likely to have higher hospital commercial-to-Medicare price ratios than markets with low hospital concentration levels. These high HHI markets, which were smaller and located in less urban areas, seem more ripe for policies aimed at targeting high hospital prices. We also found that insurer concentration was associated with lower ratios of commercial-to-Medicare payment rates for hospitals, as insurers with leverage seemed to exercise market power in negotiating with hospitals. However, we could not determine in this work whether individual and employer premium markets were higher or lower in concentrated insurer markets.

We also found that geozips with a teaching hospital, which were generally larger urban areas, had higher hospital price ratios compared with geozips without a teaching hospital. While some market concentration could be at play, teaching hospitals in these areas could be providing higher quality of care at a higher cost (eg, major teaching hospitals could be dealing with more rare diseases and patients with complex conditions, providing specialized high-cost services, and using advanced medical technology).

Efforts to promote competition, reduce market concentration, and limit high commercial payment rates could reign in health care prices and ultimately bring financial relief for households. CBO estimated that 4 policies that target the market power of hospitals and physicians—increasing antitrust enforcement, reducing hospitals’ incentives to consolidate, making it easier for physicians to change jobs, and prohibiting anticompetitive contracts between insurers and hospitals—could lead to small price reductions within the first 10 years of enactment, with larger reductions over the long term.^31^ Another policy option aimed at prices is site-neutral payment reform, which would reduce the amount Medicare pays for certain services in more expensive hospital settings, while eliminating the incentive for hospitals to employ physicians and purchase physician practices.

While major reforms such as the introduction of a public option plan and capped rates could produce sizeable savings for households, employers, and the federal government, these policies and others that aim to make meaningful reductions in health care spending would have tradeoffs in terms of reducing hospital and physician revenues and potentially affecting health care access and quality if not implemented carefully.

### Limitations

Although the database has a large and geographically diverse sample of claims data, the data do not contain all private plans in a state or substate area, nor do they represent all commercial payers in a specific area. In addition, it is possible that the services chosen do not represent the true mean price ratio of the commercial insurance allowed amount to Medicare. The true price ratio could vary by service category, or within each geographic area, in ways not measured by the particular *CPT* codes and geographic divisions that we selected for this study. Finally, this analysis was primarily descriptive, and prices may be associated with various factors that were not evaluated (eg, risk and demographic profiles of patients and concentration in nonhospital provider markets).

## Conclusions

This cross-sectional study of claims data found that private insurers’ allowed amounts were 246% of Medicare rates for hospital inpatient and outpatient services and 124% of Medicare rates for professional services, with substantial variation both across and within states. We also found that higher commercial-to-Medicare price ratios were associated with very high hospital market concentration levels, the presence of a major teaching hospital, and a higher share of the population who were uninsured. These findings could be used to identify and target specific areas more amenable to policies aimed at curbing hospital price growth.

## Notes

Supplement 1. **eTable.** Commercial-to-Medicare Price Ratios by State, 2020 to 2022-2023 **eFigure 1.** Commercial-to-Medicare Price Ratio for Professional Services by Geozip, 2022-2023 **eFigure 2.** Commercial-to-Medicare Price Ratio for Combined Hospital Services by Geozip, 2022-2023

Supplement 2.
Data Sharing Statement
