# Supplementary material for: Potential Factors Associated With Commercial-to-Medicare Relative Prices at the Substate Level
Source: JAMA Health Forum. 2025 Jul 3;6(7):e251640. doi: 10.1001/jamahealthforum.2025.1640 (PMC12232185; doi:10.1001/jamahealthforum.2025.1640)
Supplement: Supplement 1. — eTable. Commercial-to-Medicare Price Ratios by State, 2020 to 2022-2023 eFigure 1. Commercial-to-Medicare Price Ratio for Professional Services by Geozip, 2022-2023 eFigure 2. Commercial-to-Medicare Price Ratio for Combined Hospital Services by Geozip, 2022-2023 [file jamahealthforum-e251640-s001.pdf]

Supplemental Online Content

Blavin F, Holahan J. Potential factors associated with commercial-to-Medicare relative prices at the substate level. *JAMA Health Forum*. 2025;6(7):e251640. doi: 10.1001/jamahealthforum.2025.1640

eTable 1. Commercial-to-Medicare Price Ratios by State, 2020 to 2022-2023

eFigure 1. Commercial-to-Medicare Price Ratio for Professional Services by Geozip, 2022-2023

eFigure 2. Commercial-to-Medicare Price Ratio for Combined Hospital Services by Geozip, 2022-2023

This supplementary material has been provided by the authors to give readers additional information about their work

© 2025 Blavin F et al. *JAMA Health Forum*.

eTable 1. Commercial-to-Medicare Price Ratios by State, 2020 to 2022-2023

	Commercial-to-Medicare Price Ratio, Professional Services			Commercial-to-Medicare Price Ratio, Hospital Services		
	**2020**	**2022**	**Percent change**	**2020**	**2022**	**Percent change**
**US**	1.27	1.24	-2.2%	2.33	2.46	5.6%
**State**						
AK	2.14	2.09	-2.5%	4.34	5.00	15.2%
AL	1.47	0.91	-38.5%	1.58	1.49	-5.6%
AR	1.32	1.24	-5.6%	1.30	1.28	-1.6%
AZ	1.07	0.96	-9.7%	2.09	2.10	0.5%
CA	1.30	1.30	0.1%	2.64	2.95	11.8%
CO	1.28	1.21	-5.3%	3.21	3.45	7.5%
CT	1.31	1.19	-9.1%	2.22	2.26	1.9%
DC	1.22	1.23	0.6%	2.14	2.20	3.2%
DE	1.04	1.06	2.1%	2.02	2.17	7.6%
FL	1.24	1.23	-0.8%	2.38	2.54	6.7%
GA	1.46	1.41	-3.7%	2.15	2.62	21.9%
HI	1.26	1.05	-16.2%	2.73	2.75	0.8%
IA	1.54	1.38	-10.7%	1.72	1.73	0.8%
ID	1.44	1.31	-8.7%	2.71	2.65	-2.2%
IL	1.23	1.18	-3.6%	2.33	2.21	-5.1%
IN	1.06	0.99	-6.8%	2.33	2.47	6.0%
KS	1.19	1.10	-7.1%	2.01	2.01	-0.4%
KY	1.19	1.05	-11.8%	2.19	2.31	5.4%
LA	1.33	1.17	-12.4%	1.99	1.85	-7.3%
MA	1.43	1.44	0.2%	1.48	1.46	-1.0%
MD	1.01	0.98	-3.0%	1.71	1.69	-1.6%
ME	1.31	1.30	-0.3%	2.29	2.43	6.2%
MI	1.11	1.07	-4.1%	2.24	2.16	-3.7%
MN	2.14	2.06	-3.5%	2.23	2.21	-0.6%
MO	1.13	1.11	-2.4%	1.85	1.96	5.7%
MS	1.15	1.08	-5.7%	1.58	1.45	-8.3%
MT	1.44	1.37	-5.3%	2.70	2.80	3.9%
NC	1.44	1.35	-6.0%	2.61	2.68	2.7%
ND	1.87	1.76	-6.0%	3.16	3.29	4.3%
NE	1.70	1.61	-5.7%	2.30	2.32	0.7%
NH	1.45	1.35	-6.9%	3.27	2.86	-12.5%
NJ	1.15	1.14	-0.4%	1.98	2.14	8.1%
NM	1.26	1.25	-0.6%	1.68	2.96	76.6%
NV	1.17	1.08	-7.9%	1.84	2.17	18.0%
NY	1.20	1.18	-1.1%	2.66	2.74	3.1%

© 2025 Blavin F et al. *JAMA Health Forum*.

OH	1.19	1.15	-3.3%	2.07	2.09	0.9%
OK	1.10	1.01	-8.4%	1.93	1.92	-0.7%
OR	1.72	1.72	-0.1%	2.49	2.55	2.4%
PA	1.02	0.98	-3.3%	2.00	1.89	-5.2%
RI	1.23	1.13	-7.8%	2.05	1.96	-4.3%
SC	1.29	1.13	-12.7%	2.96	2.61	-11.7%
SD	1.62	1.46	-9.7%	3.62	3.57	-1.4%
TN	1.44	1.37	-5.1%	1.95	2.01	3.1%
TX	1.12	1.10	-2.2%	2.47	2.61	5.9%
UT	1.24	1.17	-5.6%	2.35	2.57	9.0%
VA	1.17	1.13	-3.5%	2.64	2.70	2.1%
VT	1.20	1.17	-2.0%	5.18	6.87	32.8%
WA	1.39	1.33	-4.5%	2.53	2.56	1.2%
WI	2.13	1.97	-7.4%	2.67	2.87	7.7%
WV	1.29	1.19	-7.8%	2.57	2.81	9.3%
WY	1.87	1.80	-3.8%	3.90	3.74	-4.0%

Source: FAIR Health National Private Insurance Claims (FH NPIC®) repository
Notes: Timeframes defined by dates of services, from January through December 2020, and from June 2022 through May 2023

© 2025 Blavin F et al. *JAMA Health Forum*.

eFigure 1. Commercial-to-Medicare Price Ratio for Professional Services by Geozip, 2022-2023

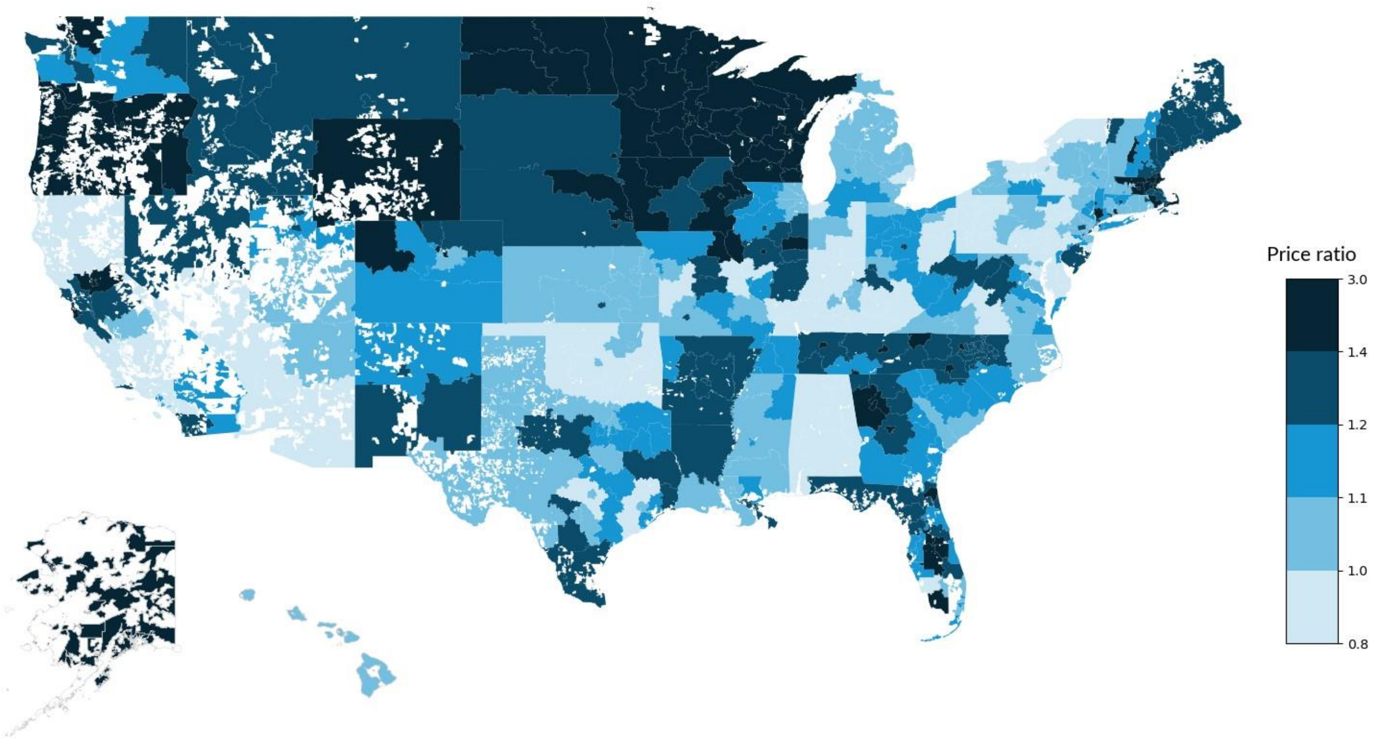

Source: FAIR Health National Private Insurance Claims (FH NPIC®) repository
Notes: Timeframe defined by dates of services from June 2022 through May 2023

© 2025 Blavin F et al. *JAMA Health Forum*.

eFigure 2. Commercial-to-Medicare Price Ratio for Combined Hospital Services by Geozip, 2022-2023

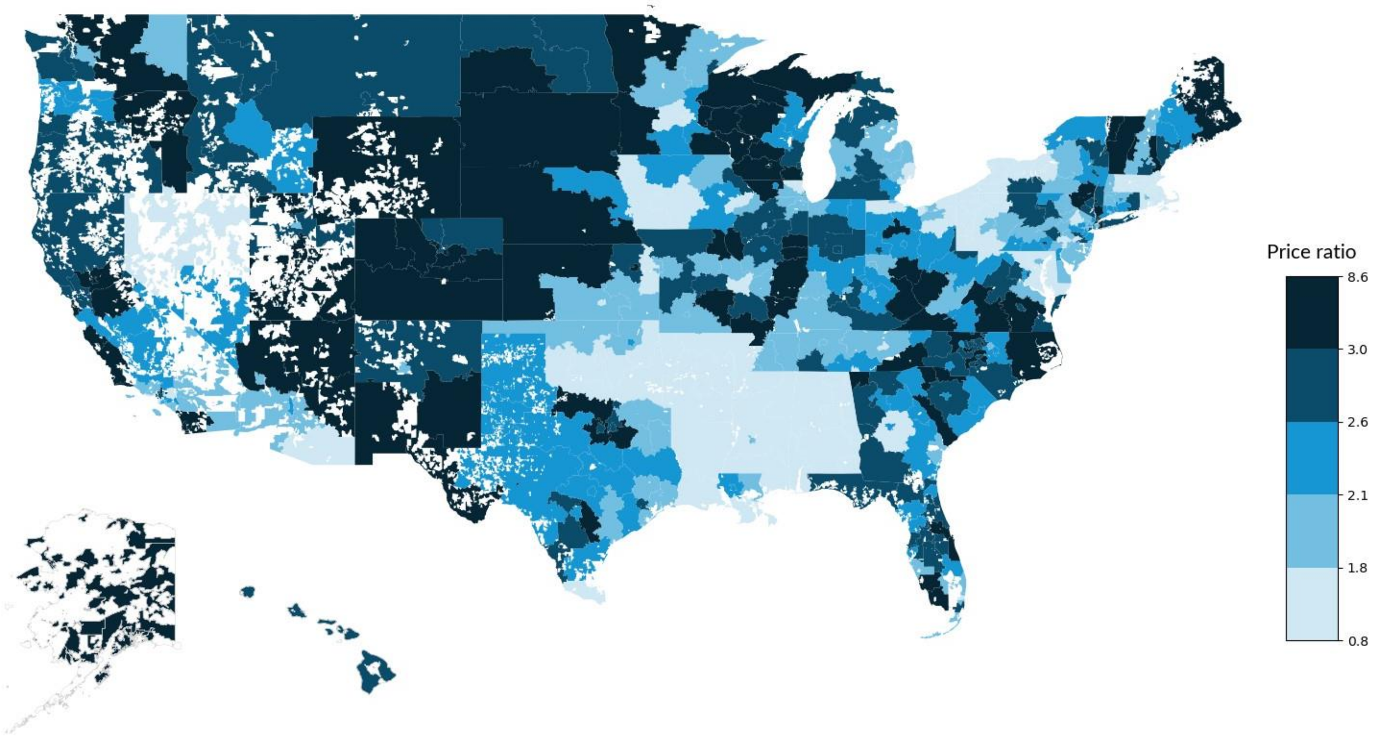

Source: FAIR Health National Private Insurance Claims (FH NPIC®) repository
Notes: Timeframe defined by dates of services from June 2022 through May 2023

© 2025 Blavin F et al. *JAMA Health Forum*.
