# Supplementary material for: Potential Factors Associated With Commercial-to-Medicare Relative Prices at the Substate Level
Source: JAMA Health Forum. 2025 Jul 3;6(7):e251640. doi: 10.1001/jamahealthforum.2025.1640 (PMC12232185; doi:10.1001/jamahealthforum.2025.1640)
Supplement: Supplement 2. — Data Sharing Statement [file jamahealthforum-e251640-s002.pdf]

Data Sharing Statement

Blavin. Potential Factors Associated With Commercial-to-Medicare Relative Prices at the Substate Level. *JAMA Health Forum*. Published July 03, 2025.
doi:10.1001/jamahealthforum.2025.1640

Data
Data available: No
